# The association between the rs6495309 polymorphism in *CHRNA3* gene and lung cancer risk in Chinese: a meta-analysis

**DOI:** 10.1038/srep06372

**Published:** 2014-10-07

**Authors:** Min Xiao, Lei Chen, Xiaoling Wu, Fuqiang Wen

**Affiliations:** 1 Division of Pulmonary Diseases, State Key Laboratory of Biotherapy of China, West China Hospital, West China School of Medicine, Sichuan University, Chengdu, China; 2 Department of Respiratory Medicine, West China Hospital, West China School of Medicine, Sichuan University, Chengdu, China

## Abstract

The association between the rs6495309 polymorphism in *CHRNA3* gene and lung cancer risk has been studied in Chinese by several number case-control control studies with small number of cases and controls, and these studies might be underpowered to reveal the true association. Thus we sought to investigate the association with the risk of lung cancer by performing a comprehensive meta-analysis on the polymorphism. Five case-control studies were extracted from 3 articles on the polymorphism involving 4608 lung cancer cases and 4617 controls. The results of meta-analysis showed that significant increased risk were found for the polymorphism with the risk of lung cancer in Chinese: OR = 1.47, 95%CI = 1.33–1.63, *P* < 0.00001 for CC + TC vs. TT; OR = 1.24, 95%CI = 1.07–1.44, *P* = 0.005 for CC vs. TT + TC; OR = 1.62, 95%CI = 1.32–2.00, *P* < 0.00001 for CC vs. TT; OR = 1.42, 95%CI = 1.26–1.61, *P* < 0.00001 for CT vs. TT; OR = 1.42, 95%CI = 1.26–1.61, *P* < 0.00001. No significant publication bias was found for the five genetic models. Our findings demonstrated that *CHRNA3* gene rs6495309 polymorphism might be a risk factor for the development of lung cancer in Chinese.

Lung cancer is one of the most common malignant tumors in humans and is the most common cause of cancer-related mortality^1^,^2^. Epidemiology studies suggested that lung cancer arises as a result of complex interactions of environmental factors^3^,^4^. Chronic smoking, occupational exposure, air pollution and other factors are risk factors for lung cancer^5^,^6^. In addition, genetic factors also contribute to the risk of lung cancer^7^. In recent years, many individual studies have set out to determine whether there is an association between genetic polymorphisms and lung cancer susceptibility, such as *CHRNA3* polymorphisms^8^,^9^. However, these studies showed conflicting results that failed to provide compelling evidence for lung cancer susceptibility.

The human *CHRNA3* gene is located on the chromosome 15q25.1 region which has been identified as a hotspot for lung cancer susceptibility by recent genome-wide association (GWA) studies^10^,^11^,^12^. Several polymorphisms in the gene have been identified, such as the rs6495309, rs8034191 and rs1051730^10^,^11^,^12^. Among these polymorphisms, the rs6495309 is one of the widely studied polymorphisms for Chinese, and several studies have performed to study the association between the polymorphism with the risk of lung cancer in Chinese^13^,^14^,^15^. Although they found the polymorphism might contribute to the risk of lung cancer in Chinese, however, the results are still needed to be further validated, because individual study with small sample sizes may be underpowered to detect the effect of CHRNA3 genotype on the susceptibility of lung cancer for Chinese. In order to get more precision results for the polymorphism and the risk of lung cancer in Chinese, we carried out a meta-analysis including all eligible studies published to date to systematically and comprehensively estimate the association the polymorphism and susceptibility to lung cancer among Chinese population. This is, to our knowledge, the first meta-analysis that investigated the association between the CHRNA3 polymorphisms and lung cancer risk for Chinese.

## Methods

### Literature search strategy

The databases of PubMed, Embase, CNKI and Wanfang were searched (the last search was updated in Feb 20^th^, 2014) to identify all relevant publications on the association between *CHRNA3* rs6495309 polymorphism and lung cancer risk in Chinese. The following search terms and their synonyms were used: lung cancer and polymorphism and *CHRNA3* and Chinese. We also manually searched the reference lists of all eligible studies and review articles to obtain additional usable data that can be included in the current meta-analysis.

### Inclusion criteria and exclusion criteria

We selected eligible studies according to the following criteria: (1) the study must have a case-control design; (2) the association between *CHRNA3* rs6495309 polymorphism and lung cancer risk must be examined in Chinese; (3) adequate genotyping data must be contained such that odds ratios (ORs) with 95% confidence intervals (CIs) could be calculated; (4) the study had to be published using human subjects. Exclusion criteria were: (1) insufficient information on the distribution of *CHRNA3* genotypes; (2) case-only studies; (3) duplicated publications. If a study was subsequently updated, we selected the study with the largest sample size. Two investigators independently reviewed all studies to examine whether they fulfilled the inclusion criteria.

### Data extraction

Two independent investigators extracted the original data according to the inclusion criteria and exclusion criteria to ensure the accuracy of the retrieved information. The data extracted from each eligible study included the first author's name, year of publication, cancer type, ethnicity, source of controls, method adopted for genotyping, number of cases and controls and genotype frequencies. Disputes were settled by consulting the third person.

### Statistical analysis

Hardy-Weinberg equilibrium (HWE) of the control groups was tested by the χ2 test for goodness of fitness. Crude ORs with 95% CIs were calculated to evaluate the strength of the association between the polymorphism and lung cancer risk. The pooled ORs were performed for the following genetic models: allele contrast (C vs. T), homozygote (CC vs. TT), heterozygote (CT vs. TT), dominant (CC + CT vs. CC) and recessive (TT vs. CT + CC) model. Heterogeneity assumption was evaluated by the chi-square based Q-test and *I^2^* statistics, *P* > 0.05 for the Q test or *I^2^* < 50% suggested a lack of heterogeneity. In this situation, the OR of each study was calculated by the fixed-effects model (the Mantel-Haenszel method). If *P* < 0.05 or *I^2^* > 50%, the random-effects model (the DerSimonian and Laird method) was used^16^,^17^. Sensitivity analysis was performed by removing one study at a time to ensure that our findings were not driven by any single study. The evaluation of potential publication bias was performed using the Begg's funnel plots and Egger's test^18^. All ORs for the five genetic models will be compared with each other, and the genetic model with the greatest OR and statistical significant result will be the inheritance model that is most likely to contribute the risk of lung cancer. All statistical analyses were performed by Revman5.2.0 and STATA11.0. A level of *P* < 0.05 was accepted as statistically significant.

## Results

### Characteristics of published studies

**Figure 1:**
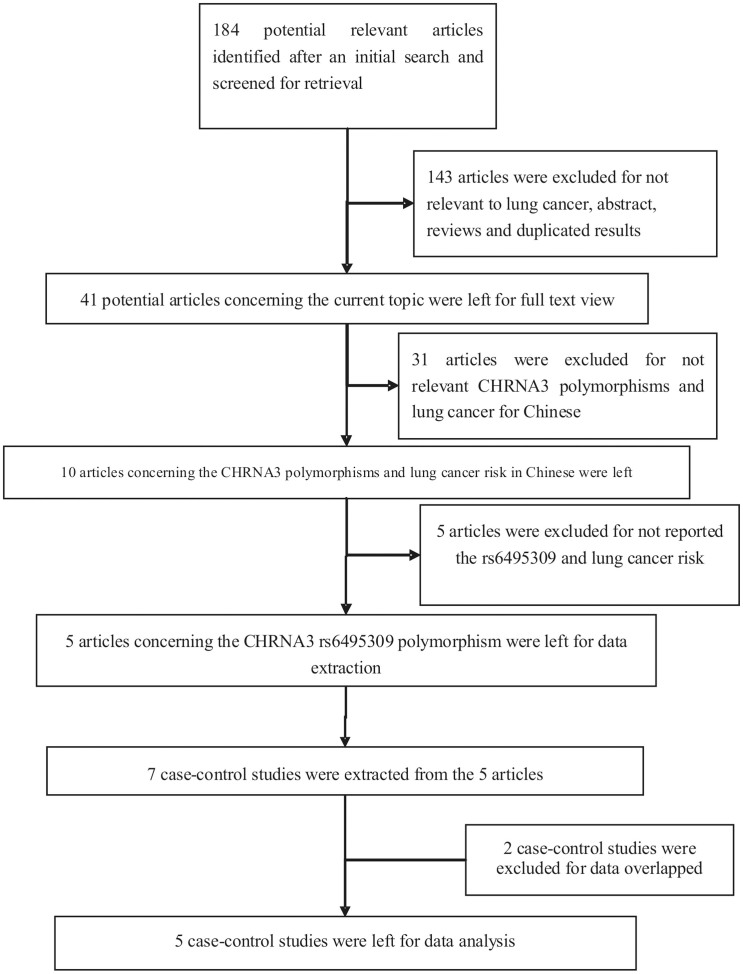
Flow chart explaining the selection of eligible studies included in the meta-analysis.

The screening of the studies is shown in Figure 1. The literature search yielded 184 articles at initial screening. After removing the articles that investigated the association between cancer and polymorphism rather than lung cancer and CHRNA polymorphisms, reviews and abstracts, 41 potential articles were left for further assessment. Further evaluation of eligibility by reviewing full texts excluded 31 publications because of not assessing Chinese. In addition, 5 studies were excluded for not analyzing the association between the rs6495309 polymorphism and lung cancer risk for Chinese. Thus, a total of 5 articles that investigated the association between the rs6495309 polymorphism in CHRNA3 gene and the risk of lung cancer in Chinese were left for data extraction. Two of these articles investigated lung cancer patients and control subjects in both two provinces and the data were analyzed separately for each group; these data were treated as independent case-control studies^19^,^20^. Thus, a total of 7 case-control studies were extracted from these 5 articles. In addition, two case-control studies were excluded for data overlapped. Finally, a total of 5 case-control studies from 3 articles were used for data analysis^19^,^20^,^21^. The characteristics of the included studies are shown in Table 1. Genotype distributions of all control groups were in accord with HWE.

### Quantitative analysis

#### CC + TC vs. TT

**Figure 2:**
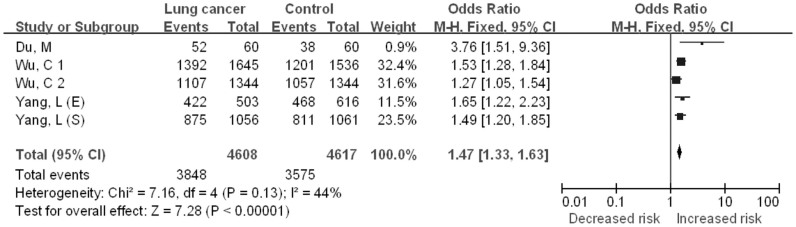
The association between the CHRNA3 rs6495309 polymorphism and the risk of lung cancer in Chinese: CC + TC vs. TT comparative.

The five case–control studies included in the quantitative analysis yielded a total of 4608 lung cancer cases and 4617 controls for the CC + CT vs. TT comparative (Figure 2). No significant between-study heterogeneity was detected across studies for the CC + TC vs. TT model and thus we selected the fix-effects model to summarize the ORs. Overall, we found a significant association between *CHRNA3* rs6495309 polymorphism and lung cancer risk for Chinese (OR = 1.47, 95%CI = 1.33–1.63, *P* < 0.00001). Begg's funnel plots and Egger's test were performed to evaluate publication bias in the literature. Funnel plots of the genetic model seemed symmetrical (Figure not shown). This was confirmed by the statistical data derived using Egger's test (*t* = 2.46, *P* = 0.091).

#### CC vs. TT + TC

**Figure 3:**
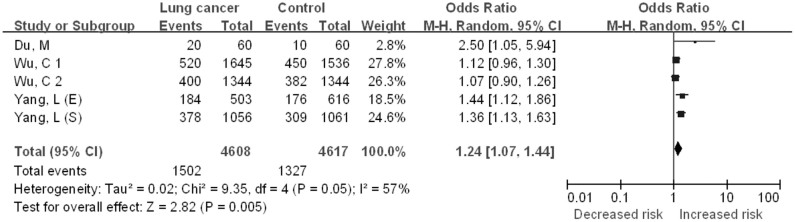
The association between the *CHRNA3* rs6495309 polymorphism and the risk of lung cancer in Chinese: CC vs. TT + TC comparative.

The five case–control studies included in the quantitative analysis yielded a total of 4608 lung cancer cases and 4617 controls for the CC vs. TT + CT comparative (Figure 3). Significant between-study heterogeneity was detected across studies for the CC vs. TT + CT model and thus we selected the random-effects model to summarize the ORs. Overall, we found a significant association between *CHRNA3* rs6495309 polymorphism and lung cancer risk for Chinese (OR = 1.24, 95%CI = 1.07–1.44, *P* = 0.005). Begg's funnel plots and Egger's test were performed to evaluate publication bias in the literature. Funnel plots of the genetic model seemed symmetrical (Figure not shown). This was confirmed by the statistical data derived using Egger's test (*t* = 2.08, *P* = 0.129).

#### CC vs. TT

**Figure 4:**
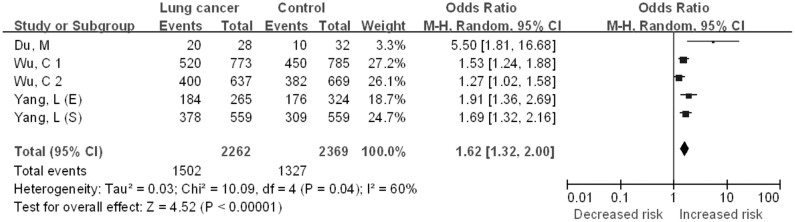
The association between the *CHRNA3* rs6495309 polymorphism and the risk of lung cancer in Chinese: CC vs. TT comparative.

The five case–control studies included in the quantitative analysis yielded a total of 2262 lung cancer cases and 2369 controls for the CC vs. TT comparative (Figure 4). Significant between-study heterogeneity was detected across studies for the CC vs. TT model and thus we selected the random-effects model to summarize the ORs. Overall, we found a significant association between *CHRNA3* rs6495309 polymorphism and lung cancer risk for Chinese (OR = 1.62, 95%CI = 1.32–2.00, *P* < 0.00001). Begg's funnel plots and Egger's test were performed to evaluate publication bias in the literature. Funnel plots of the genetic model seemed symmetrical. This was confirmed by the statistical data derived using Egger's test (*t* = 2.68, *P* = 0.075).

#### CT vs. TT

**Figure 5:**
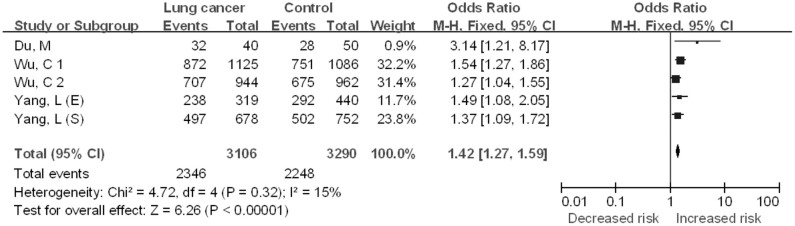
The association between the *CHRNA3* rs6495309 polymorphism and the risk of lung cancer in Chinese: CT vs. TT comparative.

The five case–control studies included in the quantitative analysis yielded a total of 3106 lung cancer cases and 3290 controls for the CT vs. TT comparative (Figure 5). No significant between-study heterogeneity was detected across studies for the CT vs. TT model and thus we selected the fixed-effects model to summarize the ORs. Overall, we found a significant association between *CHRNA3* rs6495309 polymorphism and lung cancer risk for Chinese (OR = 1.42, 95%CI = 1.26–1.61, *P* < 0.00001). Begg's funnel plots and Egger's test were performed to evaluate publication bias in the literature. Funnel plots of the genetic model seemed symmetrical. This was confirmed by the statistical data derived using Egger's test (*t* = 1.74, *P* = 0.181).

#### C vs. T

**Figure 6:**
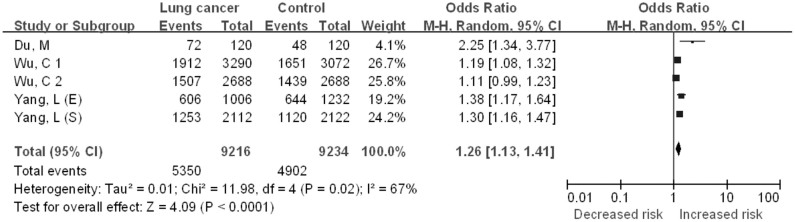
The association between the *CHRNA3* rs6495309 polymorphism and the risk of lung cancer in Chinese: C vs. T comparative.

The five case–control studies included in the quantitative analysis yielded a total of 4608 lung cancer cases and 4617 controls for the C vs. T comparative (Figure 6). Significant between-study heterogeneity was detected across studies for the C vs. T model and thus we selected the random-effects model to summarize the ORs. Overall, we found a significant association between *CHRNA3* rs6495309 polymorphism and lung cancer risk for Chinese (OR = 1.26, 95%CI = 1.13–1.41, *P* < 0.0001). Begg's funnel plots and Egger's test were performed to evaluate publication bias in the literature. Funnel plots of the genetic model seemed symmetrical. This was confirmed by the statistical data derived using Egger's test (*t* = 2.79; *P* = 0.069).

## Discussion

Via a comprehensive meta-analysis, we evaluated the association of one common polymorphism in the *CHRNA3* gene with the risk of lung cancer for Chinese. Although potential sources of heterogeneity could not be easily eliminated, the present study, to our knowledge, is the first meta-analysis to date dealing with the association of the rs6495309 polymorphism with lung cancer susceptibility for Chinese.

In this meta-analysis, we included a total of five case-control studies. The pooled results indicated that there were obvious associations between *CHRNA3* rs6495309 polymorphism and lung cancer in Chinese under all models: allele contrast (C vs. T), homozygote (CC vs. TT), heterozygote (CT vs. TT), dominant (CC + CT vs. CC) and recessive (TT vs. CT + CC) model. Thus, the *CHRNA3* rs6495309 polymorphism could be suggested as a lung cancer risk factor for Chinese.

Although previous case-control studies suggested the rs6495309 polymorphism might contribute to the risk of lung cancer for Chinese, however, these studies were with small number of cases and controls. Thus, we performed the current meta-analysis. In the current meta-analysis, the results showed that the value of the OR for CC *vs.* TT is the largest among all five ORs; thus, the individuals who carries the CC homozygote might have more increased risks. Previously, Wu *et al.* reported^19^ that the rs6495309T/C change would considerably influence the CHRNA3 promoter activity, and resulting in significant increase in the CHRNA3 RNA expression with rs6495309 C allele compared with the rs6495309T allele^19^. These effects might be resulted from reduced ability of the rs6495309 C allele to bind Oct-1, a transcriptional factor that has been shown to repress gene transcription^22^. In addition, the CHRNA3 was associated with more consume cigarettes, and leading to more damage in pulmonary function, and would be expected to be at higher risk for developing lung cancer^20^. Taken together, the results of CC vs. TT inheritance model is consistent with previous biological results, because, individual carried the variant homozygote CC may affect more for reducing ability to bind the repress transcriptional factor Oct-1, and thus increase the expression of CHRNA3, and thus increase the risk of lung cancer.

Meta-analysis is a useful method for investigating associations of diseases with genetic factors because it uses a quantitative approach by way of combing the results of different studies on the same topic, and potentially providing more conclusive results^23^. Recently, accumulated meta-analyses have been published for the investigating the association of genetic variants and diseases^24^,^25^,^26^,^27^. Some meta-analysis investigated only one polymorphism with the risk of disease, while some analyzed more than one polymorphism, this might be dependent on the number of original case-control studies. If the included case-control studies for one polymorphism was one or two, there is no needed to perform a meta-analysis. As for the CHRNA3 gene, despite of the rs6495309 polymorphism, several polymorphisms were also investigated for the associations with lung cancer risk for Chinese, such as the rs8034191 and rs1051730. However, the included case-control studies for these polymorphisms were so small; thus, we did not assess these two polymorphisms in the current meta-analysis. And we did not assess the interaction between different polymorphisms of the gene with the risk of lung cancer. In future, if there are more case-control studies for these polymorphisms, we will update our meta-analysis.

As far as we know, there has been one published meta-analyses regarding *CHRNA3* polymorphism and lung risk^28^. According to Gu et al.(2012)^28^, significant association between *CHRNA3* rs1051730 polymorphism and lung cancer was acquired. Compared with the previous meta-analysis, we added another CHRNA3 polymorphism that might contribute lung cancer risk in Chinese using newly published studies.

Several limitations should be acknowledged when interpreting the results of this meta-analysis. First, there was a potential language bias, because the PubMed, EMbase, CNKI and Wanfang search engines were used to identify articles and to exclude articles written in languages other than English and Chinese. This might not have prevented the researchers from accessing all relevant studies. Second, relatively few eligible studies, all with small sample sizes, were included in this meta-analysis, which could increase the risk of random error. To conduct a more precise analysis of *CHRNA3* rs6495309 polymorphism and the risk of lung cancer, further investigations with larger sample sizes and higher quality are needed. Third, the overall outcomes were based on individual unadjusted ORs; a more precise estimation should be adjusted by menstrual status, age, environmental and other confounding factors. Finally, this meta-analysis could not address the gene-gene and gene-environmental interactions in the association between *CHRNA3* rs6495309 polymorphism and lung cancer risk. Future studies that include detailed information on exposures to various carcinogens and individual-level data to assess the possible gene-gene and gene-environment interactions in the association between *CHRNA3* rs6495309 polymorphism and lung cancer risk are needed.

This meta-analysis of five case-control studies suggested that *CHRNA3* rs6495309 polymorphism is associated with an increased risk of lung cancer in Chinese. Additional studies with a greater number of patients should be performed to examine how the *CHRNA3* variants interact with other risk loci to influence lung cancer risk.

## Author Contributions

F.W. designed the study, wrote the manuscript. M.X., L.C. and X.W. performed the analyses. All authors reviewed the manuscript.

## Figures and Tables

**Table 1:** The characteristics of the included case-control studies

			Lung cancer			Control			
Study	Year	Location	TT	TC	CC	TT	TC	CC	HWE
Du, M^21^	2011	Shandong	8	32	20	22	28	10	Yes
Wu, C 1^19^	2009	Beijing	253	872	520	335	751	450	Yes
Wu, C 2^19^	2009	Jiangsu	237	707	400	287	675	382	Yes
Yang, L (E)^20^	2012	Jiangsu	81	238	184	148	292	176	Yes
Yang, L (S)^20^	2012	Guangdong	181	497	378	250	502	309	Yes
